# Effects of vaccinia virus uracil DNA glycosylase catalytic site and deoxyuridine triphosphatase deletion mutations individually and together on replication in active and quiescent cells and pathogenesis in mice

**DOI:** 10.1186/1743-422X-5-145

**Published:** 2008-12-02

**Authors:** Frank S De Silva, Bernard Moss

**Affiliations:** 1 Laboratory of Viral Diseases, National Institute of Allergy and Infectious Diseases, National Institutes of Health, Bethesda, Maryland 20892-3210, USA; 2 Scientific Review Program, NIAID, NIH, 6700B Rockledge Dr., Bethesda, MD 20892-7616, USA

## Abstract

**Background:**

Low levels of uracil in DNA result from misincorporation of dUMP or cytosine deamination. Vaccinia virus (VACV), the prototype poxvirus, encodes two enzymes that can potentially reduce the amount of uracil in DNA. Deoxyuridine triphosphatase (dUTPase) hydrolyzes dUTP, generating dUMP for biosynthesis of thymidine nucleotides while decreasing the availability of dUTP for misincorporation; uracil DNA glycosylase (UNG) cleaves uracil N-glycosylic bonds in DNA initiating base excision repair. Studies with actively dividing cells showed that the VACV UNG protein is required for DNA replication but the UNG catalytic site is not, whereas the dUTPase gene can be deleted without impairing virus replication. Recombinant VACV with an UNG catalytic site mutation was attenuated *in vivo*, while a dUTPase deletion mutant was not. However, the importance of the two enzymes for replication in quiescent cells, their possible synergy and roles in virulence have not been fully assessed.

**Results:**

VACV mutants lacking the gene encoding dUTPase or with catalytic site mutations in UNG and double UNG/dUTPase mutants were constructed. Replication of UNG and UNG/dUTPase mutants were slightly reduced compared to wild type or the dUTPase mutant in actively dividing cells. Viral DNA replication was reduced about one-third under these conditions. After high multiplicity infection of quiescent fibroblasts, yields of wild type and mutant viruses were decreased by 2-logs with relative differences similar to those observed in active fibroblasts. However, under low multiplicity multi-step growth conditions in quiescent fibroblasts, replication of the dUTPase/UNG mutant was delayed and 5-fold lower than that of either single mutant or parental virus. This difference was exacerbated by 1-day serial passages on quiescent fibroblasts, resulting in 2- to 3-logs lower titer of the double mutant compared to the parental and single mutant viruses. Each mutant was more attenuated than a revertant virus upon intranasal infection of mice.

**Conclusion:**

VACV UNG and dUTPase activities are more important for replication in quiescent cells, which have low levels of endogenous UNG and dUTPase, than in more metabolically active cells and the loss of both is more detrimental than either alone. Both UNG and dUTPase activities are required for full virulence in mice.

## Background

Uracil, a major component of RNA, is a rare constituent of DNA due to misincorporation of dUMP from dUTP or the spontaneous deamination of cytosine residues [1]. The presence of uracil in DNA can have adverse effects. U:A pairs arising from misincorporation are not mutagenic per se since they can be corrected in the next round of replication. However, U:G mispairs arising from deamination would lead to transition mutations. Free-living organisms as well as some viruses encode uracil DNA glycosylase (UNG) and deoxyuridine 5'-triphosphate (dUTPase), which may lower the amount of uracil in DNA [2,3]. By hydrolyzing dUTP, dUTPase generates dUMP for the biosynthesis of thymidine nucleotides while concurrently decreasing the availability of dUTP for misincorporation [4]. UNG specifically recognizes uracil in DNA and initiates base excision repair by hydrolyzing the glycosylic bond linking uracil to a deoxyribose sugar. An abasic site is created that is removed by a 5'-acting apurinic/apyrimidic endonuclease and a DNase, leaving a gap filled by DNA polymerase and sealed by ligase [5].

Viruses that encode UNG or dUTPase include poxviruses, herpesviruses, African swine fever virus and some retroviruses [3]. Poxviruses are large, complex viruses that reside exclusively in the cytoplasm of host cells and encode DNA polymerase and other enzymes and factors necessary to replicate their double-stranded DNA genomes [6,7]. All sequenced members of the chordopoxvirus subfamily encode an UNG [8-10]. Because cellular UNGs have a repair function that is unnecessary for viability, it was surprising that vaccinia virus (VACV) UNG encoded by the D4R (VACV-WR-109) open reading frame is essential for DNA replication [10,11]. Subsequent mutagenesis studies, however, showed that the critical role of D4 (the protein encoded by D4R) is independent of its DNA glycosylase activity, though the latter is required for full virulence in a mouse intranasal infection model [12]. D4 has a direct role in replication as it is complexed with other viral replication proteins and increases the processivity of the DNA polymerase [13-16]. Many poxviruses, including variola virus and VACV, encode a dUTPase as well as an UNG [17]. In contrast to UNG, however, the dUTPase encoded by the VACV F2L (VACV-WR-041) open reading frame was deleted without affecting viral replication in cell culture or virulence in a mouse infection model, although hypersensitivity to the drug (N)-methanocarbathymidine suggests that pyrimidine metabolism is altered in infected cells [18,19]. African swine fever virus, distantly related to poxviruses, encodes a dUTPase that is dispensable for replication in dividing Vero cells but is required for efficient replication in non-dividing swine macrophages [20].

Herpesviruses are large, DNA viruses that replicate in the nucleus and encode UNG and dUTPase as well as DNA polymerase [21-23]. The UNG proteins encoded by herpes simplex virus type 1 (HSV-1) and varicella zoster virus are dispensable for replication in cultured cells [22,24], but are required for efficient HSV-1 replication and latent infection in the murine nervous system [25]. Deletion of the cytomegalovirus UNG caused a delay in viral DNA replication in quiescent human fibroblasts [26,27]. It was suggested that UNG creates sites in cytomegalovirus DNA that are used for recombination-dependent replication late in infection. HSV-1 dUTPase is also dispensable for replication in actively growing cultured cells but is required for full neurovirulence in a mouse model [25,28].

Although retroviruses do not encode UNG, HIV-1 packages a cellular UNG that is essential for its life cycle [29-31]. Interestingly, the packaging of a heterologous UTPase can complement the defect associated with the absence of HIV-1 virion-associated UNG [31]. β-retroviruses and non-primate lentiviruses encode a dUTPase that is important for replication in non-dividing macrophages and inducing disease [32-35].

Taking together the results from a variety of systems, it seems that the requirements for virus-encoded UNG and dUTPase are greatest in quiescent cells [3], which have low endogenous UNG and dUTPase levels [36,37] and relatively high ratios of dUTP to dTTP [38,39]. In addition, we considered that there might be a greater need for UNG if dUTP levels increased in the absence of dUTPase. For the present study, we compared the replication of single and double VACV dUTPase deletion and UNG catalytic site mutants in actively growing and quiescent cells as well as the virulence of the mutants in a mouse respiratory infection model.

## Results

### Construction of single and double VACV UNG and dUTPase mutants

**Figure 1:**
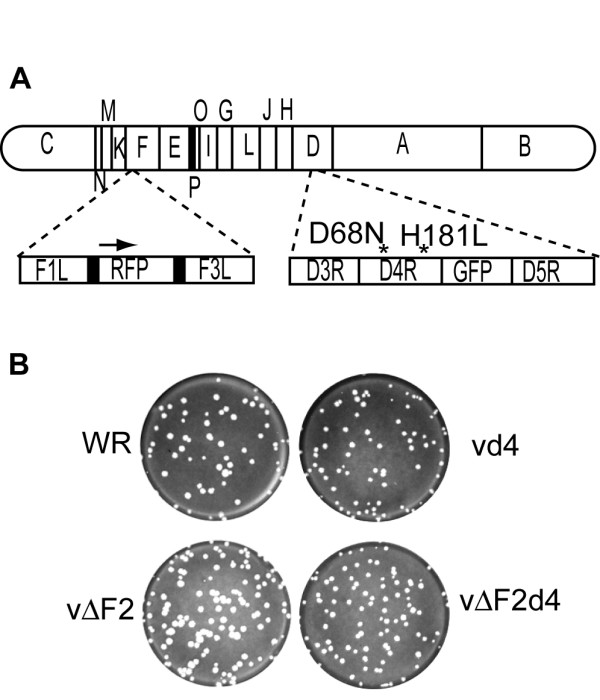
**Construction and plaque formation of VACV mutants with deletion of the dUTPase gene and active site mutations in the uracil DNA glycosylase gene**. (A) Schematic diagram of the VACV genome organization. The WR genome is represented with expansions showing replacement of F2L gene with RFP in vΔF2 and vΔF2d4 and D68N and H181L point mutations (indicated by asterisks) in D4R gene with adjacent green fluorescent protein gene in vd4 and vΔF2d4. (B) Plaques of WR, vd4, vΔF2, and vΔF2d4 on BS-C-1 cells. Viruses were plated on BS-C-1 cell monolayers and covered with a semi-solid methylcellulose overlay. After 2 days at 37°C, the monolayers were stained with crystal violet.

The VACV D4R catalytic site mutant with Asp-68-Asn and His-181-Leu changes and containing an enhanced green fluorescent protein (GFP) reporter gene was previously constructed and shown to lack DNA glycosylase activity [12]. This D4R catalytic site mutant will be referred to here as vd4. The F2L gene was deleted from both wild type VACV WR (referred to subsequently as WR) and from vd4 by recombination with a plasmid containing red fluorescent protein (RFP) flanked by F1L and F3L DNA to form vΔF2 and vΔF2d4, respectively (Fig. 1A). WR and vΔF2 plaques on BS-C-1 cells were indistinguishable, whereas those of vd4 and vΔF2d4 were slightly smaller (Fig. 1B) as had been noted previously for vd4 [12]. Thus, deletion of the F2L gene did not alter the plaque size of WR or further reduce the plaque size of vd4.

### Replication of mutated viruses and DNA in actively dividing BS-C-1 cells

**Figure 2:**
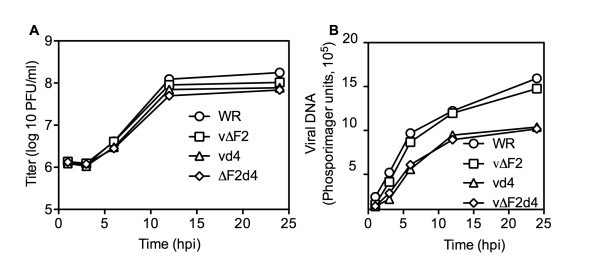
**Virus replication and DNA synthesis in BS-C-1 cells**. Confluent 6 well plates of BS-C-1 cells were infected with WR and recombinant viruses at a multiplicity of 5. The infected cells were harvested at the indicated hours post infection (hpi) to determine virus titers and viral DNA. (A) Virus titers were determined on BS-C-1 cells and plotted as PFU/ml. All experiments were carried out in triplicate. Average titers are shown; bars representing standard error of the mean could not be printed because of their very close spacing. (B) Viral DNA was determined by blot hybridization and quantified with a phosphorImager. Experiment represents average of duplicate experiments.

Our previous study showed that vd4 yielded slightly lower titers than WR in RK13 cells under one-step growth conditions and this correlated with slightly lower levels of DNA synthesis [12]. Here, we compared the replication kinetics of the D4 and F2 single and double mutant viruses in BS-C-1 cells. The 24 h titers were consistently WR > ΔF2 > vd4 ~ΔF2d4, though the differences were very small (Fig. 2A).

To measure viral DNA synthesis, BS-C-1 cells were infected with WR or mutant viruses at a multiplicity of 5 plaque-forming units (PFU) per cell. At various times, infected cells were harvested and total DNA was isolated. Viral DNA accumulation was quantified by hybridization to a ^32^P-labeled VACV DNA probe. The kinetics of vd4 and vΔF2d4 DNA replication were virtually identical and the amounts of DNA were about one-third lower than VACV WR at 24 h (Fig. 2B). In contrast, vΔF2 DNA synthesis was close to that of WR at all time points (Fig. 2B).

### Replication of mutated viruses in active and resting human foreskin fibroblasts (HFF)

**Figure 3:**
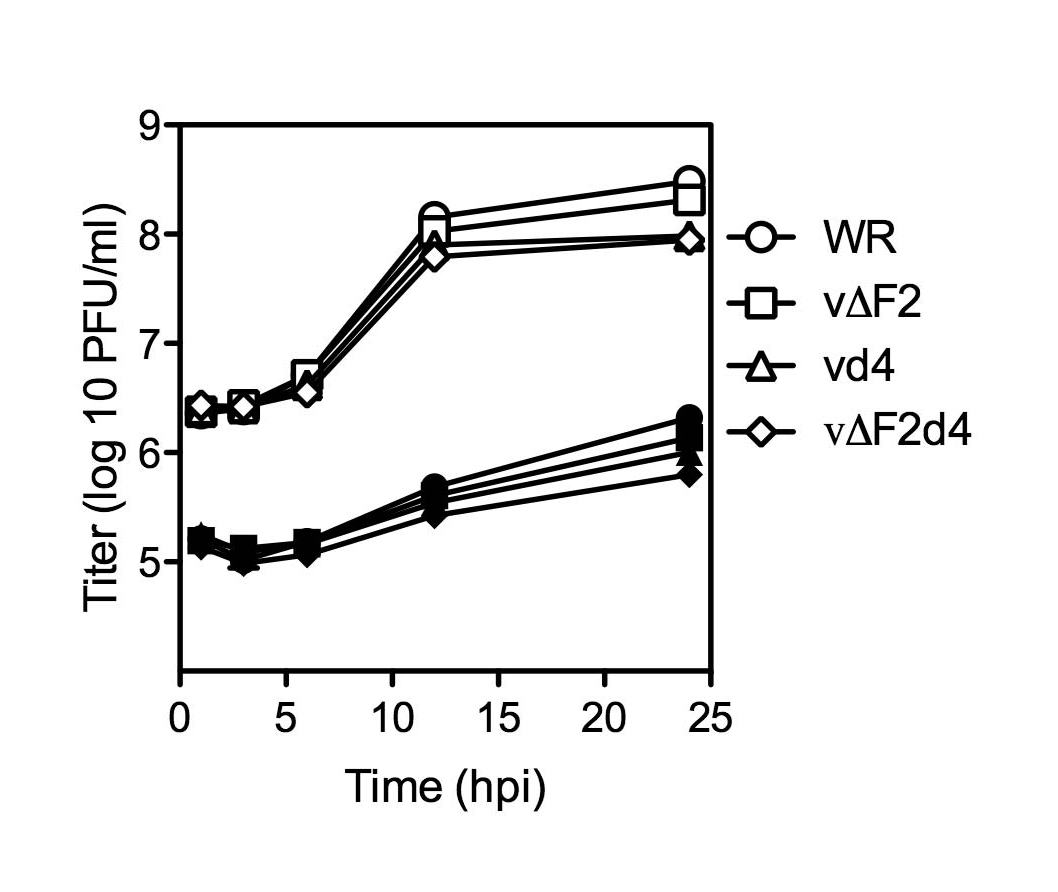
**One-step virus growth in metabolically active and quiescent HFF**. Confluent 6-well plates of active (open symbols) and quiescent (closed symbols) HFF were infected with WR and recombinant viruses at a multiplicity of 5. The infected cells were harvested at the indicated times post infection and virus titers were determined on BS-C-1 cells. Average titers are shown; bars representing standard error of the mean could not be printed because of their very close spacing.

The relatively modest effect of the UNG and dUTPase mutations could be a consequence of the presence of the corresponding cellular enzymes in actively replicating cells. Therefore, we compared the replication of the mutated viruses in actively growing HFF propagated in 10% fetal bovine serum (FBS) or in stationary cells that had been incubated for 4 days in 0.2% FBS. Active and quiescent HFF were infected with WR or mutant viruses at a multiplicity of 5 and harvested at sequential times. As with BS-C-1 cells, vd4 and vΔF2d4 replicated to slightly lower titers than WR and vΔF2 in active HFF (Fig. 3). In quiescent HFF, the recoveries of input viruses at 1 h were approximately 1 log less than for the active HFF, suggesting reduced virus attachment (Fig. 3). Virus titers slowly increased between 6 and 24 h but were still about 2-logs less than in the metabolically active cells (Fig. 3). Nevertheless, by 12 h most cells appeared to be expressing fluorescent protein, although less brightly than in active cells. The relative differences in yields between VACV and mutant viruses were similar in resting and active cells.

**Figure 4:**
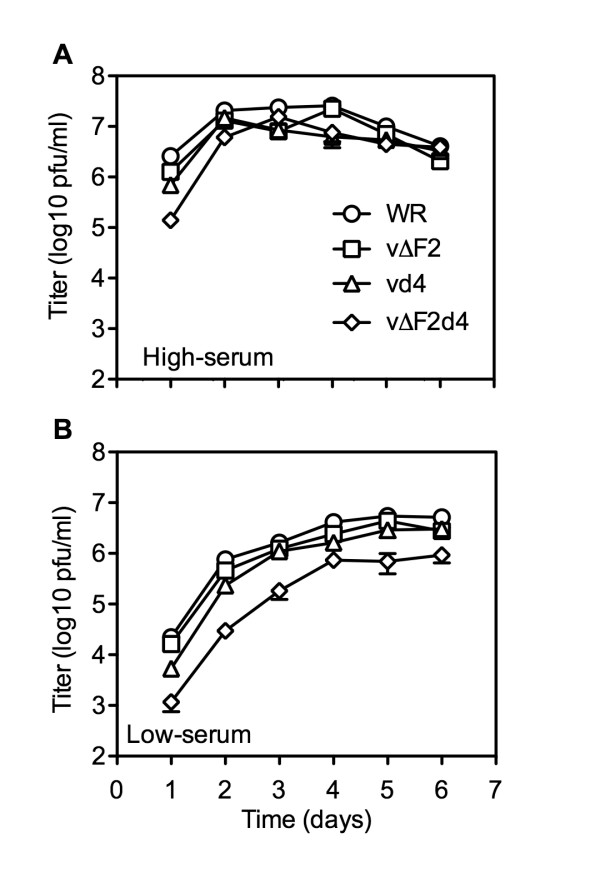
**Low multiplicity virus infection and spread in active and quiescent HFF**. Confluent 6 well plates of active (A) and quiescent (B) HFF were infected with viruses at a multiplicity of 0.001. Every 24 h triplicate wells were harvested and titered on BS-C-1 cells. Average titers are shown. Error bars represent the standard error of the mean.

Phenotypic differences between mutant viruses can be more pronounced when cells are infected at a low multiplicity, in which virus spread is also assessed. Therefore, we infected HFF at a multiplicity of 0.001 and measured virus replication over a 6-day period. At one day after infection of active HFF, the relative titers were WR > vΔF2 > vd4 > vΔF2d4 (Fig. 4A). In each case the virus titers increased by day 2 but then reached a plateau and decreased. By days 5 and 6, the titers of the different viruses were similar (Fig. 4A). In the quiescent cells, virus titers increased for 4 to 5 days and except for vΔF2d4 eventually reached titers similar to the final titers in the actively growing cells (Fig. 4B). On days 2 and 6, the titers of vΔF2d4 were 25- and 5.5-fold lower, respectively, than WR. All infected monolayers, except those inoculated with vΔF2d4, showed extensive cytopathic effects by day 5.

**Figure 5:**
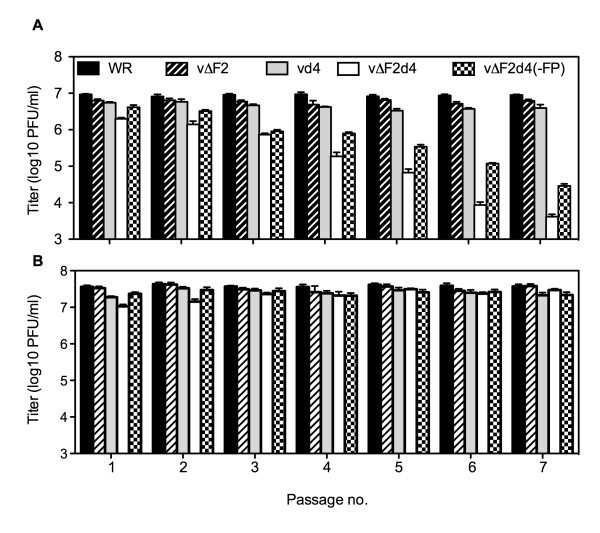
**Serial virus passage in active and quiescent HFF**. Quiescent (A) and active (B) HFF were infected with 5 PFU per cell of WR or indicated mutant virus. After 24 h, the cells were harvested and 10% of the lysate used to infect fresh quiescent or active HFF. The procedure was repeated for a total of 7 serial infections. Experiments were carried out in triplicate. Average titers are shown and bars represent the standard error of the mean.

VACV expresses a growth factor called VGF that is secreted from infected cells and is important for replication in resting cells [40]. Therefore, secreted VGF may have stimulated the metabolism of the HFF cells in low serum over time contributing to the ability of vΔF2d4 to partly catch up to WR. To reduce this effect, we developed a protocol in which active and quiescent HFF were initially infected at a multiplicity of 5. Then, each day the cells were harvested, washed, resuspended in fresh medium, lysed and 10% used to inoculate new active or quiescent cells. In addition, we wanted to rule out the possibility that the replication of vΔF2d4 was in some way compromised by the expression of both GFP and RFP. Therefore, a new recombinant virus vΔF2d4(-FP) was made by sequentially deleting the two reporter genes from vΔF2d4. The titers of vΔF2d4 and vΔF2d4(-FP) decreased by 2- to 3-logs over a period of 7 passages in the resting cells (Fig. 5A), whereas the titers were maintained in actively growing cells (Fig. 5B). The vd4 and vΔF2 titers were only slightly less than those of WR, with a maximum 2.2-fold difference.

### Mutant viruses are less pathogenic than wild-type virus

We had previously reported that vd4 was attenuated compared to WR in a murine intranasal infection model [12]. Here we compared the virulence of vd4 to vΔF2 and vΔF2d4. As an additional control to rule out a spontaneous mutation affecting virulence that might have occurred elsewhere in the genome during the construction of the recombinant viruses, we made a revertant virus vΔF2d4rev in which the dUTPase gene and the unmutated UNG gene of vΔF2d4 were restored with the simultaneous deletion of the two fluorescent reporter genes. vΔF2d4rev formed normal size plaques and replicated like wild type virus (data not shown).

**Figure 6:**
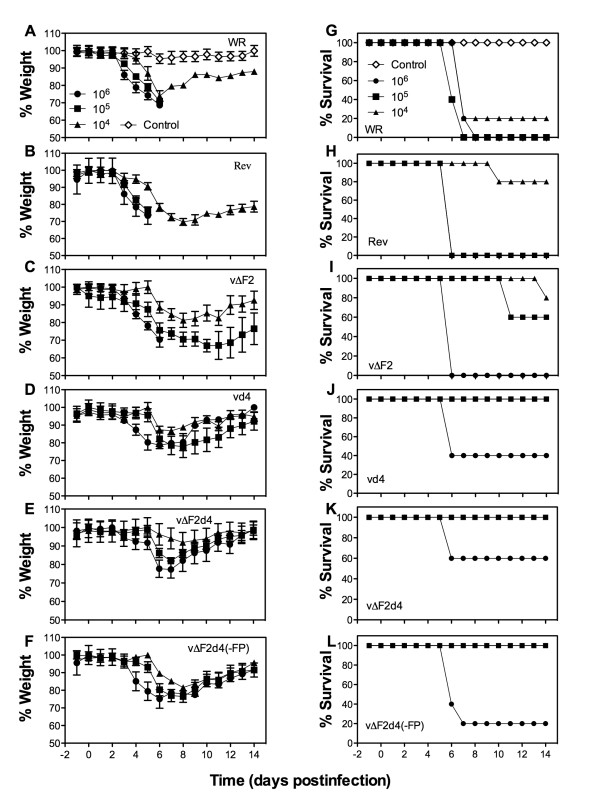
**Weight loss and lethality following intranasal infections**. Six-week female BALB/c mice were infected intranasally with 10^4^-10^6 ^PFU of purified WR or mutant or revertant (Rev) viruses. Mice were weighed daily and animals that lost ≥ 30% of their original weight were terminated. The % average weight of each group at the indicated times was plotted (A-F). Graphs represent mean of experiment (n = 5 mice/group) and bars represent the standard error of the mean. (G-L) The % of surviving mice at each time was plotted.

Groups of Balb/c mice were infected intranasally with 10^4 ^to 10^6 ^PFU of each virus and loss of weight was followed for two weeks. All animals in the WR and revertant groups that had been infected with 10^5 ^or 10^6 ^PFU died or were terminated by day 6 because their weights dropped by 30% (Fig. 6A, B, G, H). Of the mice infected with 10^4 ^PFU of WR or revertant, one and four animals survived, respectively, consistent with the LD_50 _of approximately 10^4^. Mice infected with vΔF2 did better; although those inoculated with 10^6 ^PFU died, all inoculated with 10^4 ^or 10^5 ^PFU survived for at least 10 days and the majority for 14 days (Fig. 6C, I). Mice infected with vd4, vΔF2d4, or vΔF2d4(-FP) did still better as all survived infections with 10^4 ^and 10^5 ^PFU and some even survived 10^6 ^PFU (Fig. 6D–F, J–L). The statistical significance of the differences in weight loss on day 5, prior to any deaths, was determined (Table 1). The difference between the revertant virus and each of the mutants was significant, whereas the differences between mutants were mostly not significant.

**Table 1:** Statistical significance of weight loss between infected groups of mice

Comparison	Titer	Day	No. mice	P-value	P < 0.05
vΔF2d4rev versus vΔF2	10^4^	5	5	0.0079	Yes
	
	10^5^	5	5	0.0079	Yes
	
	10^6^	5	5	0.8413	No

vΔF2d4rev versus vd4	10^4^	5	5	0.0079	Yes
	
	10^5^	5	5	0.0079	Yes
	
	10^6^	5	5	0.6905	No

vΔF2d4rev versus vΔF2d4	10^4^	5	5	0.0317	Yes
	
	10^5^	5	5	0.0079	Yes
	
	10^6^	5	5	0.0079	Yes

vΔF2d4rev versus vΔF2d4(-FP)	10^4^	5	5	0.0079	Yes
	
	10^5^	5	5	0.0079	Yes
	
	10^6^	5	5	0.0952	No

vΔF2 versus vd4	10^4^	5	5	0.0952	No
	
	10^5^	5	5	0.1508	No
	
	10^6^	5	5	0.5476	No

vΔF2 versus vΔF2d4	10^4^	5	5	0.3095	No
	
	10^5^	5	5	0.0556	No
	
	10^6^	5	5	0.0079	Yes

vΔF2 versus vΔF2d4(-FP)	10^4^	5	5	0.3095	No
	
	10^5^	5	5	0.8413	No
	
	10^6^	5	5	0.1508	No

vd4 versus vΔF2d4	10^4^	5	5	0.5476	No
	
	10^5^	5	5	0.5476	No
	
	10^6^	5	5	0.1508	No

vd4 versus vΔF2d4(-FP)	10^4^	5	5	0.1508	No
	
	10^5^	5	5	0.0317	Yes
	
	10^6^	5	5	0.8413	No

P values of all surviving mice (n = 5) on day 5 at specific viral titers were calculated based on the Mann-Whitney Test (non-parametric, two-tailed, and 95% confidence intervals) with P < 0.05 considered significant.

## Discussion

The molecular structures and catalytic activities of VACV UNG and dUTPase have been well characterized [9,17,41-44], but less is known about their roles in virus replication. Indeed, the conservation of these enzymes in poxviruses contrasted with the apparent unimportance of the virus encoded UNG and dUTPase activities for replication in tissue culture cells [12,19]. These observations were confirmed in the present study where we found that even double mutants with a catalytic site mutation in UNG and deletion of dUTPase exhibited only a small defect in replication in actively growing tissue culture cells. However, active cells have higher endogenous levels of dUTPase and UNG [36,37] and lower levels of dUTP [38,39] than resting cells. Presumably for this reason, other viruses with dUTPase or UNG deletions have a more debilitated phenotype in resting cells than active cells [3,26,27]. In the present study, we found that mutation of VACV dUTPase or UNG had a relatively small growth effect in quiescent human fibroblasts but that mutation of both caused a large decrease in replication.

Previous studies had shown that VACV replicates more poorly in resting mouse 3T3 cells than actively growing cells and this was most severe in low multiplicity, multicycle infections with a mutant virus unable to express the secreted growth factor VGF [40]. With wild type virus, secreted VGF could activate resting cells prior to the next round of infection. The restriction in resting 3T3 cells was not determined, although it appeared to be mainly a post-entry phenomenon. In contrast, wild type VACV is unable to bind to and enter resting T lymphocytes [45]. For the present study we used stationary cultures of primary human fibroblasts that were maintained for four days in low serum. Under one-cycle growth conditions, the 24 h yields of virus were wild-type > dUTPase mutant > UNG mutant > double mutant with about 1/2 log difference between the highest and lowest. However, even the wild type VACV titers were about a log lower in quiescent compared to active fibroblasts. This general effect appeared to be partly due to decreased binding since the difference was seen in the first time points prior to replication. However, this was only part of the story since most cells were infected by 12 h. The disparity between the replication of the double mutant and the other mutants and wild type virus was more marked under low multiplicity, multicycle infection conditions. On day two, the difference was about 25-fold but only 5-fold on day six. We considered that the mutant might be catching up with time because of the secretion of VGF. Therefore, we altered the protocol so that each day the cells were harvested and a new culture of cells were inoculated. Under these conditions, the difference in resting cells between the double mutant and parental virus was 2 – 3 logs by day seven. In contrast, the difference between the single mutants and parental virus was only 2-fold.

In quiescent cells, the impairment caused by deletion of the dUTPase gene could have resulted from decreased availability of dUMP for biosynthesis of thymidine nucleotides or from misincorporation of uracil nucleotides due to increased amounts of dUTP. The impairment caused by mutation of UNG, could have resulted from the failure to excise uracil and resulting transition mutations. However, we could not confirm the presence of excess uracil in DNA purified from dUTPase/UNG double mutants. Following treatment with UNG, we found 44 and 38 apurinic sites per 100,000 base pairs in parental and mutant DNA, respectively (FDS, unpublished data). The possibility of an increased mutation rate needs to be investigated.

The intranasal mouse model has been extensively used to determine virulence of mutant VACV and morbidity and death results primarily from the respiratory infection [46-51]. We reproduced our previous finding that the VACV UNG catalytic site mutant was attenuated in this model [12]. In addition, we found that mice inoculated with the dUTPase mutant or the double mutant lost less weight and survived higher doses of virus than those inoculated with a revertant virus. However, we did not find a statistically significant difference in the attenuation of the single and double mutants. In contrast to our results, Prichard and coworkers [19] did not find that a VACV dUTPase deletion mutant was attenuated. The difference might be due to the greater susceptibility to VACV infection of the 3-week old mice used in the previous study compared to the 6-week old mice used here. It would be interesting to examine other routes of virus inoculation since the replication of the mutant viruses may be cell type dependent.

## Conclusion

VACV recombinants with mutations in the catalytic site of UNG and/or a deletion of the dUTPase gene were constructed. In actively growing cells, the UNG mutant and the double mutant exhibited a slight reduction in replication, whereas replication of the single dUTPase deletion mutant was unimpaired. However, in quiescent human fibroblasts, which have low levels of endogenous UNG and dUTPase, replication of the double mutant was more severely inhibited than either of the single mutants. Expression of viral UNG and dUTPase were required for full virulence in mice.

## Methods

### Cells, virus, and plasmids

HFF were obtained from A. McBride (NIAID, NIH, Bethesda, MD). Monolayer cultures of HeLa S3, HFF and BS-C-1 cells were maintained in Eagle's minimal essential medium (EMEM; Quality Biologicals, Inc, Gaithersburg, MD) containing L-glutamine and 10% FBS. All experiments were performed with the WR strain of VACV (ATCC VR-1354) or with mutant viruses derived from this strain. The VACV D4R catalytic site mutant containing Asp-68-Asn and His-181-Leu changes was previously described [12]. Plasmid pUC19slp containing a VACV synthetic late promoter was provided by C. Ansarah-Sobrinho (NIAID, NIH, Bethesda, MD). Plasmid pslpRed was created by cloning the RFP open reading frame by PCR using Accuprime pfx (Invitrogen, Carlsbad, CA) and primers 5'-ACCATGGTGAGCGGCCTGCTGAAGGAG-3' and 5'-TTAGTTGGCCTTCTCGGGCAGGTCGCTGTA-3', then digesting the PCR product with Sal I/BamH I followed by ligation of the digested product between the Sal I/BamH I sites of pUC19slp. Plasmid pΔF2 containing RFP flanked by F1 and F3 sequences was constructed as follows: DNA containing the F1L and 76 nucleotides of F2L was obtained by PCR using Accuprime pfx and primers 5'-CCGGAATTCCTTACACCCAACCCCTTGTTATCCA-3' and 5'-CCGGAATTCTCCAGAACTGGAAGAAGTACAATCTCT-3'.

The PCR product was inserted into an EcoR I site upstream of the RFP gene. A segment of DNA containing F3L and 74 nucleotides of F2L using Accuprime pfx and primers 5'-AAAACTGCAGATGCTGCTTGGGTTAATATGCCGAGT-3' and 5'-CGCGGATCCTGCCTAGTAGGAGATTTAGCTCTGT-3' was amplified by PCR. The PCR product was inserted between BamH I and Pst I sites downstream of the RFP gene. The general procedures used for preparing and titrating the viral stocks were described previously [52].

### Construction of VACV F2L deletion and D4R catalytic site mutants

Approximately 10^6 ^BS-C-1 cells were infected with WR or vd4 at a multiplicity of 0.05 PFU per cell for 1 h at 37°C. The infected cells were washed twice with Opti-MEM (Invitrogen) and transfected with 2 μg of pΔF2. After 5 h, the transfection mixture was replaced with EMEM/2.5% FBS, and the cells were harvested at 48 h in 0.5 ml of EMEM/2.5% FBS. Lysates were prepared by freezing and thawing the cells three times and sonicating them twice for 30 s. Recombinant viruses that expressed RFP (vΔF2) or both RFP and GFP (vΔF2d4) were plaque purified five times on BS-C-1 cells and their genetic purity was confirmed by PCR, Southern blotting, and sequencing.

To construct recombinant vΔF2d4 (-FP) lacking both GFP and RFP genes, PCR products were made by overlapping PCR that had (i) F1 and F3 sequences but missing the F2L ORF and (ii) D4 and D5 sequences that maintained the catalytic site mutations mutations of D4. 

F1r: 5'-CCGCTCGAGCGGTTACACCCAACCCCTTGTTATCCATTAG-3', 

F1f: 5'-CTAACAGAGCTAAATCTCCTACTATCCAGAACTGGAAGAAGTACAATCTCTA-3', 

F3r: 5'-TTGTACTTCTTCCAGTTCTGGATAGTAGGAGATTTAGCTCTGTTAGTTTCC-3', 

F3f: 5'-CCCAAGCTTGGGATGCTGCTTGGGTTAATATGCCGAGTC-3', 

D4f: 5'-CCGCTCGAGCGGATGAATTCAGTGACTGTATCACACGCGCC-3', 

D4r: 5'-TTAGAACACAAGTTAAAATTTCACTAAAGGTTAATAAATAAACCCTTGAGCCCAATTTAT-3',

 D5f: 5'-CTTTAGTGAAATTTTAACTTGTGTTCTAAATGGATGCGGCTATTAGAGGTAATGATG-3', 

D5r: 5'-CCCAAGCTTGGGTTTCTCCTATATACGGCAGTGTCTATCG-3'. 

The procedure for making recombinants was the same as above, except vΔF2d4 was used to infect BS-C-1 cells and virus that lacked RFP was first selected (i.e. only green fluorescence) followed by virus that lacked RFP and GFP (no fluorescence).

To construct a revertant that has wild type F2L and D4R sequences without RFP and GFP, PCR products were made that had (i) F1-F2-F3 sequences and (ii) D4-D5 sequences from WR. Primers F1r and F3f were used to amplify DNA from WR. Primers P1: 5'-CGAGTATGTGTGTGTGGTATAGATCC-3' and P2: 5'-CGGCAGTGTCTATCGATCTTGTTAGTG-3' were used to amplify DNA from WR. Virus was constructed as above using vΔF2d4 and virus that lacked RFP was first selected (i.e. only green fluorescence) followed by virus that lacked RFP and GFP (no fluorescence).

### One-step virus growth

For one step virus growth, confluent BS-C-1 or HFF in six-well plates were infected with 5 PFU of virus per cell and maintained at 37°C for 1 h. The innocula were removed, cells washed three times, and overlaid with 2 ml of EMEM/2.5% FBS. The cells were maintained at 37°C and harvested at various times after infection, collected by centrifugation, and resuspended in EMEM/2.5% FBS. The cells were disrupted by three cycles of freezing and thawing and two 30-s bursts of sonication. Virus yields were determined by titration on BS-C-1 cells.

### Detection of viral DNA by slot blot hybridization

Confluent BS-C-1 cells in six-well plates were infected with 5 PFU of virus per cell. The cells were collected by centrifugation, washed once with phosphate-buffered saline, and re-suspended in 0.3 ml of 1.5 M NaCl-0.15 M sodium citrate (pH 7.0)-1 M ammonium acetate. The cells were disrupted by three cycles of freezing and thawing. After dispersal by vortexing, duplicate 50 μl samples were spotted onto an Immobilon-Ny+ transfer membrane in a slot blot apparatus. The membrane was treated sequentially with 0.5 M NaOH-1 M Tris-HCl (pH 7.5) and 0.3 M NaCl-0.03 M sodium citrate (pH 7.0) and cross-linked by UV irradiation. The membrane was air dried, and viral DNA was detected by hybridization using a radiolabeled probe generated by random priming using VACV I7 gene nucleotide sequences, Random labeling kit (Invitrogen, Carlsbad, CA) and [α-^32^P]dCTP (PerkinElmer, Shelton, CT). The blot was exposed to a phosphor screen, and data were acquired on a Storm PhosphorImager (Molecular Dynamics, Sunnyvale, CA) and quantified with ImageQuant software (Molecular Dynamics).

### Infection of active and quiescent HFF

HFF were seeded into 6 well tissue culture plates in EMEM/10% FBS. When monolayers were confluent, the culture medium was replaced with EMEM/0.2% FBS and maintained under these conditions for 96 h to induce quiescence. Following this treatment, HFF were infected with recombinant virus in EMEM/0.2% FBS. Alternatively, cells were propagated in EMEM/10% FBS and as soon as confluent the active cell monolayers were infected with recombinant virus in EMEM/2.5% FBS. At the indicated times post infection, infected monolayers were harvested and resuspended in EMEM/2.5% FBS. The cells were disrupted by three cycles of freezing and thawing and two 30-s bursts of sonication. Virus yields were determined by titration on BS-C-1 cells.

### Animal studies

Purified virus for animal studies were obtained as follows. HeLa cells (2.5 × 10^8^) were infected with 3 PFU of virus per cell and harvested three days after infection. Cells were re-suspended in 10 mM Tris (pH 9.0) and Dounce homogenized. The cytoplasm was separated from nuclei by low speed centrifugation and layered on a 36% sucrose cushion. After centrifugation at 13,500 rev/min in a SW 28.1 rotor for 80 min at 4°C, the viral pellet was suspended in 10 mM Tris (pH 9.0) and the virus titer determined by plaque assay. Groups (n = 5) of 6-week-old, female BALB/c mice were anesthetized and inoculated intranasally with 10^4^-10^6 ^PFU of virus in 20 μl (half in each nostril). Each mouse was weighed daily and animals that lost 30% of their original body weight were terminated according to a protocol approved by the NIAID Animal Care and Use Committee.

### Statistical Methods

Significance was determined with the Mann-Whitney test (non-parametric, two-tailed, and 95% confidence) using Prism (Graphpad Software, La Jolla, Ca).

## Competing interests

The authors declare that they have no competing interests.

## Authors' contributions

FDS participated in the design and coordination of the study, acquisition and analysis of data, and preparation of the manuscript. BM designed and coordinated the study, assisted in the data analyses and contributed to the preparation of the manuscript.

## References

[B1] Sousa MM, Krokan HE, Slupphaug G (2007). DNA-uracil and human pathology. Mol Aspects Med.

[B2] Krokan HE, Drablos F, Slupphaug G (2002). Uracil in DNA – occurrence, consequences and repair. Oncogene.

[B3] Chen R, Wang H, Mansky LM (2002). Roles of uracil-DNA glycosylase and dUTPase in virus replication. J Gen Virol.

[B4] Kornberg RD, Lorch Y (1992). Chromatin structure and transcription. Annu Rev Cell Biol.

[B5] Krokan HE, Nilsen H, Skorpen F, Otterlei M, Slupphaug G (2000). Base excision repair of DNA in mammalian cells. FEBS Lett.

[B6] Moss B, De Silva F, DePamphilis ML (2006). Poxvirus DNA replication and human disease. DNA Replication & Human Disease.

[B7] Moss B, Knipe DM, Howley PM (2007). Poxviridae: the viruses and their replication. Fields Virology.

[B8] Upton C, Stuart DT, McFadden G (1993). Identification of a poxvirus gene encoding a uracil DNA glycosylase. Proc Natl Acad Sci USA.

[B9] Stuart DT, Upton C, Higman MA, Niles EG, McFadden G (1993). A poxvirus-encoded uracil DNA glycosylase is essential for virus viability. J Virol.

[B10] Millns AK, Carpenter MS, DeLange AM (1994). The vaccinia virus-encoded uracil DNA glycosylase has an essential role in viral DNA replication. Virology.

[B11] Holzer G, Falkner FG (1997). Construction of a vaccinia virus deficient in the essential DNA repair enzyme uracil DNA glycosylase by a complementing cell line. J Virol.

[B12] De Silva FS, Moss B (2003). Vaccinia virus uracil DNA glycosylase has an essential role in DNA synthesis that is independent of its glycosylase activity: catalytic site mutations reduce virulence but not virus replication in cultured cells. J Virol.

[B13] McCraith S, Holtzman T, Moss B, Fields S (2000). Genome-wide analysis of vaccinia virus protein-protein interactions. Proc Natl Acad Sci USA.

[B14] Stanitsa ES, Arps L, Traktman P (2006). Vaccinia virus uracil DNA glycosylase interacts with the A20 protein to form a heterodimeric processivity factor for the viral DNA polymerase. J Biol Chem.

[B15] Ishii K, Moss B (2002). Mapping interaction sites of the A20R protein component of the vaccinia virus DNA replication complex. Virology.

[B16] Ishii K, Moss B (2001). Role of vaccinia virus A20R protein in DNA replication: construction and characterization of temperature-sensitive mutants. J Virol.

[B17] Broyles SS (1993). Vaccinia virus encodes a functional dUTPase. Virology.

[B18] Perkus ME, Goebel SJ, Davis SW, Johnson GP, Norton EK, Paoletti E (1991). Deletion of 55 open reading frames from the termini of vaccinia virus. Virology.

[B19] Prichard MN, Kern ER, Quenelle DC, Keith KA, Moyer RW, Turner PC (2008). Vaccinia virus lacking the deoxyuridine triphosphatase gene (F2L) replicates well in vitro and in vivo, but is hypersensitive to the antiviral drug (N)-methanocarbathymidine. Virol J.

[B20] Oliveros M, Garcia-Escudero R, Alejo A, Vinuela E, Salas ML, Salas J (1999). African swine fever virus dUTPase is a highly specific enzyme required for efficient replication in swine macrophages. J Virol.

[B21] Caradonna SJ, Cheng YC (1981). Induction of uracil-DNA glycosylase and dUTP nucleotidohydrolase activity in herpes simplex virus-infected human cells. J Biol Chem.

[B22] Mullaney J, Moss HW, McGeoch DJ (1989). Gene UL2 of herpes simplex virus type 1 encodes a uracil-DNA glycosylase. J Gen Virol.

[B23] Preston VG, Fisher FB (1984). Identification of the herpes simplex virus type 1 gene encoding the dUTPase. Virology.

[B24] Reddy SM, Williams M, Cohen JI (1998). Expression of a uracil DNA glycosylase (UNG) inhibitor in mammalian cells: varicella-zoster virus can replicate in vitro in the absence of detectable UNG activity. Virology.

[B25] Pyles RB, Thompson RL (1994). Evidence that the herpes simplex virus type 1 uracil DNA glycosylase is required for efficient viral replication and latency in the murine nervous system. J Virol.

[B26] Prichard MN, Duke GM, Mocarski ES (1996). Human cytomegalovirus uracil DNA glycosylase is required for the normal temporal regulation of both DNA synthesis and viral replication. J Virol.

[B27] Courcelle CT, Courcelle J, Prichard MN, Mocarski ES (2001). Requirement for uracil-DNA glycosylase during the transition to late-phase cytomegalovirus DNA replication. J Virol.

[B28] Fisher FB, Preston VG (1986). Isolation and characterisation of herpes simplex virus type 1 mutants which fail to induce dUTPase activity. Virology.

[B29] Willetts KE, Rey F, Agostini I, Navarro JM, Baudat Y, Vigne R, Sire J (1999). DNA repair enzyme uracil DNA glycosylase is specifically incorporated into human immunodeficiency virus type 1 viral particles through a Vpr-independent mechanism. J Virol.

[B30] Mansky LM, Preveral S, Selig L, Benarous R, Benichou S (2000). The interaction of vpr with uracil DNA glycosylase modulates the human immunodeficiency virus type 1 In vivo mutation rate. J Virol.

[B31] Priet S, Gros N, Navarro JM, Boretto J, Canard B, Querat G, Sire J (2005). HIV-1-associated uracil DNA glycosylase activity controls dUTP misincorporation in viral DNA and is essential to the HIV-1 life cycle. Mol Cell.

[B32] Lichtenstein DL, Rushlow KE, Cook RF, Raabe ML, Swardson CJ, Kociba GJ, Issel CJ, Montelaro RC (1995). Replication in vitro and in vivo of an equine infectious anemia virus mutant deficient in dUTPase activity. J Virol.

[B33] Lerner DL, Wagaman PC, Phillips TR, Prospero-Garcia O, Henriksen SJ, Fox HS, Bloom FE, Elder JH (1995). Increased mutation frequency of feline immunodeficiency virus lacking functional deoxyuridine-triphosphatase. Proc Natl Acad Sci USA.

[B34] Steagall WK, Robek MD, Perry ST, Fuller FJ, Payne SL (1995). Incorporation of uracil into viral DNA correlates with reduced replication of EIAV in macrophages. Virology.

[B35] Turelli P, Guiguen F, Mornex JF, Vigne R, Querat G (1997). dUTPase-minus caprine arthritis-encephalitis virus is attenuated for pathogenesis and accumulates G-to-A substitutions. J Virol.

[B36] Ladner RD, Caradonna SJ (1997). The human dUTPase gene encodes both nuclear and mitochondrial isoforms. Differential expression of the isoforms and characterization of a cDNA encoding the mitochondrial species. J Biol Chem.

[B37] Muller SJ, Caradonna S (1993). Cell cycle regulation of a human cyclin-like gene encoding uracil-DNA glycosylase. J Biol Chem.

[B38] Traut TW (1994). Physiological concentrations of purines and pyrimidines. Mol Cell Biochem.

[B39] Aquaro S, Calio R, Balzarini J, Bellocchi MC, Garaci E, Perno CF (2002). Macrophages and HIV infection: therapeutical approaches toward this strategic virus reservoir. Antiviral Res.

[B40] Buller RML, Chakrabarti S, Moss B, Frederickson T (1988). Cell proliferative response to vaccinia virus is mediated by VGF. Virology.

[B41] Roseman NA, Evans RK, Mayer EL, Rossi MA, Slabaugh MB (1996). Purification and characterization of the vaccinia virus deoxyuridine triphosphatase expressed in Escherichia coli. J Biol Chem.

[B42] Schormann N, Grigorian A, Samal A, Krishnan R, DeLucas L, Chattopadhyay D (2007). Crystal structure of vaccinia virus uracil-DNA glycosylase reveals dimeric assembly. BMC Struct Biol.

[B43] Samal A, Schormann N, Cook WJ, DeLucas LJ, Chattopadhyay D (2007). Structures of vaccinia virus dUTPase and its nucleotide complexes. Acta Crystallogr D Biol Crystallogr.

[B44] Duraffour S, Ishchenko AA, Saparbaev M, Crance JM, Garin D (2007). Substrate specificity of homogeneous monkeypox virus uracil-DNA glycosylase. Biochemistry.

[B45] Chahroudi A, Chavan R, Koyzr N, Waller EK, Silvestri G, Feinberg MB (2005). Vaccinia virus tropism for primary hematolymphoid cells is determined by restricted expression of a unique virus receptor. J Virol.

[B46] Turner GS (1967). Respiratory infection of mice with vaccinia virus. J Gen Virol.

[B47] Hayasaka D, Ennis FA, Terajima M (2007). Pathogeneses of respiratory infections with virulent and attenuated vaccinia viruses. Virol J.

[B48] Lee SL, Roos JM, McGuigan LC, Smith KA, Cormier N, Cohen LK, Roberts BE, Payne LG (1992). Molecular attenuation of vaccinia virus: mutant generation and animal characterization. J Virol.

[B49] Luker KE, Hutchens M, Schultz T, Pekosz A, Luker GD (2005). Bioluminescence imaging of vaccinia virus: Effects of interferon on viral replication and spread. Virology.

[B50] Kettle S, Blake NW, Law KM, Smith GL (1995). Vaccinia virus serpins B13R (SPI-2) and B22R (SPI-1) encode *M*_r _38.5 and 40K, intracellular polypeptides that do not affect virus virulence in a murine intranasal model. Virology.

[B51] Parkinson JE, Smith GL (1994). Vaccinia virus gene A36R encodes a Mr 43–50 K protein on the surface of extracellular enveloped virus. Virology.

[B52] Earl PL, Cooper N, Wyatt LS, Moss B, Carroll MW, Ausubel FM, Brent R, Kingston RE, Moore DD, Seidman JG, Smith JA, Struhl K (1998). Preparation of cell cultures and vaccinia virus stocks. Current Protocols in Molecular Biology.

